# Reactome Pengine: a web-logic API to the *Homo sapiens* reactome

**DOI:** 10.1093/bioinformatics/bty181

**Published:** 2018-03-30

**Authors:** Samuel R Neaves, Sophia Tsoka, Louise A C Millard

**Affiliations:** 1 Department of Informatics, King's College London, Strand, London, UK; 2 Population Health Sciences, Bristol Medical School, University of Bristol, Bristol, UK; 3 MRC Integrative Epidemiology Unit (IEU), University of Bristol, Bristol, UK; 4 Intelligent Systems Laboratory, Department of Computer Science, University of Bristol, Bristol, UK

## Abstract

**Summary:**

Existing ways of accessing data from the Reactome database are limited. Either a researcher is restricted to particular queries defined by a web application programming interface (API) or they have to download the whole database. Reactome Pengine is a web service providing a logic programming-based API to the human reactome. This gives researchers greater flexibility in data access than existing APIs, as users can send their own small programs (alongside queries) to Reactome Pengine.

**Availability and implementation:**

The server and an example notebook can be found at https://apps.nms.kcl.ac.uk/reactome-pengine. Source code is available at https://github.com/samwalrus/reactome-pengine and a Docker image is available at https://hub.docker.com/r/samneaves/rp4/.

**Supplementary information:**

Supplementary data are available at *Bioinformatics* online.

## 1 Introduction

Reactome (Fabregat *et al.*, 2016) is a web service that includes a database of the molecular details of cellular processes and is a leading tool for bioinformaticans working with biological pathways. Currently, users access data in Reactome using either HTML (the website), a REST API, a SPARQL API or by downloading the complete dataset for local processing. The APIs provide a convenient way to access the data but restrict this access to a set of predefined API calls, whereas downloading the complete dataset means that the data can be processed exactly as required. This work presents a tool—Reactome Pengine—that allows the flexibility of the latter, with the convenience of the former. This makes queries more efficient, saving both bandwidth and storage space, and is achieved using the logic programming language, Prolog. Logic programming is a paradigm for computer programming, where knowledge is represented in a restricted form of first order logic, as a set of facts and rules called a knowledge base (Clocksin and Mellish, 2003). The knowledge base is interrogated with queries, which are powerful due to inbuilt procedures that use the facts and rules together to infer solutions. Logic programming has much potential in bioinformatics (Angelopoulos and Wielemaker 2017; Mungall, 2009) and has been used with Reactome to build predictive models of disease (Neaves *et al.*, 2016).

## 2 Implementation

Recently, a library for building web servers using SWI-Prolog (Wielemaker *et al.*, 2012) called Pengines (Lager and Wielemaker, 2014) has been developed. Pengines allows data providers to make their Prolog knowledge base available to users via a web service [that uses a web-logic API (Lager and Wielemaker, 2014)], accessed as if it was on the user’s machine. In addition, users can send programs to the pengine to manipulate the data as they wish. This is very different from the traditional way of accessing data, where a user is either constrained to the set of queries defined in a (non-logical) web API or has to download a dataset in bulk. Pengine services support federated queries, similar to SPARQL, but with Turing complete programs executing on remote services rather than SQL-like queries. See Supplementary Material Sections 1 and 2 for more details.

The Reactome Pengine tool presented here uses the Pengine library to make a Prolog knowledge base, built on Reactome data, available to researchers on the internet. The mainstay of the knowledge base are facts retrieved from the Reactome *HomoSapiens.owl* Resource Description Framework (RDF) file, which contains circa 1.35 million RDF triples. In addition, we have also provided an intuitive set of data access predicates (which are similar to functions in other programming paradigms) that sit on top of the RDF data. These define relations between Reactome entities and provide a higher level abstraction of the data. Users can query the RDF directly or use this abstraction layer (or both) or make their own abstraction of the data. Our abstraction includes predicates that represent reactions as nodes on a graph, with edges between the nodes in the following two cases. First, an edge exists when an output of a reaction is an input of another reaction. This edge type we name *precedes*. Second, an edge exists when an output of a reaction *r*_1_ is a control of another reaction *r*_2_, and the particular edge type depends on how the output of *r*_1_ controls *r*_2_ (e.g. activation or inhibition, or subtypes of these). An example predicate that relates two reactions via a linking entity is ridReaction_ridLink_type_ridReaction/4. We also provide predicates with indexed (therefore fast) access to a set of queries that we expect to be useful for researchers, but that are computationally intensive (and hence slow without indexing). For example, ridPathway_reactions/2 relates pathways to the complete list of biochemical reaction IDs.

The Pengine library has inbuilt mechanisms to ensure the integrity of the server on which it is hosted. Security is ensured by allowing only ‘safe’predicates to be run on the Pengine server. Upon running a query the service first checks that the query is safe and returns an error if this is not the case. For example, sending a program that calls shell/1 would result in an error (because a user could for example send a shutdown command to the server). The Pengine library also contains a number of methods to manage resource allocation on the server, including restricting request execution time and the maximum number of requests that can be executed simultaneously. For more details see (Lager and Wielemaker, 2014). Finally, the service runs inside a Docker container, which isolates the service from the underlying machine, and facilitates scaling and load balancing to meet demand.

Queries to Reactome Pengine are logged such that over time we can augment the inbuilt predicates with the popular queries and programs and also build new indexes to improve performance and functionality as the service is used. This also means we can explore the possibility of applying machine learning on the collected programs to automatically learn predicates that are useful for users. Documentation describing the logical API is available at: https://apps.nms.kcl.ac.uk/reactome-pengine/. See Supplementary Material Section 2 for a full comparison of Reactome Pengine with existing approaches to accessing Reactome.

## 3 Interfacing with the Reactome Pengine

There are two main ways to access Reactome Pengine. First, the Reactome Pengine can be accessed using a web application, such as SWISH web notebooks (Wielemaker *et al.*, 2015). As with notebooks of other languages (such as Jupyter for Python), a SWISH notebook includes exectuable code interweaved with text explanations—ideal when wishing to share code with other researchers or work collaboratively. Furthermore, accessing Reactome Pengine via a notebook means that the researcher does not need to set up SWI-Prolog on their machine. SWISH includes graphical renderers such as C3 for simple chart generation and Graphviz for graph visualization. Users can also include Javascript and R code in their SWISH notebook. For example, the Javascript D3 library can be used to generate interactive visualizations. R can be used to perform statistical analyses and plot results. An example notebook demonstrating these capabilities is available at https://apps.nms.kcl.ac.uk/reactome-pengine/.

The second main way to access Reactome Pengine is within a SWI-Prolog program running on a local machine. We can do this by adding the directive ‘:-use_module(library(pengines)).’ and using the pengine_rpc/3 predicate. This can be useful for writing local application pipelines that need to access Reactome data. The output of the Reactome Pengine request can then be used as input to the next step in the pipeline (see Supplementary Material Section 3). This is especially useful where data cannot be processed in the cloud due to regulatory constraints.

In addition to these two recommended Prolog-based access options, it is also possible to access the service with other languages that support HTTP such as JavaScript Node.js. See Supplementary Material Section 3 for details including an example JavaScript function that calls Reactome Pengine.

## 4 Example usage

The accompanying SWISH notebook presents a set of interactive examples illustrating how Reactome Pengine can be queried and results presented using online tools. We now describe two particular use case examples both using SWI-Prolog 7.7. The first example in Command line interaction 1 shows a simple interactive Prolog session with two queries. The first query imports the Pengine library and the second query calls the pengine_rpc/2 predicate. The pengine_rpc/2 predicate has two arguments: (i) the server address of Reactome Pengine and (ii) the query we wish to run on Reactome Pengine. In this example, we issue the query rid_name(‘Protein1’, Name), to find the common name for the Reactome identifier ‘Protein1’. The result obtained is the variable Name, bound to ‘Rnf111’.


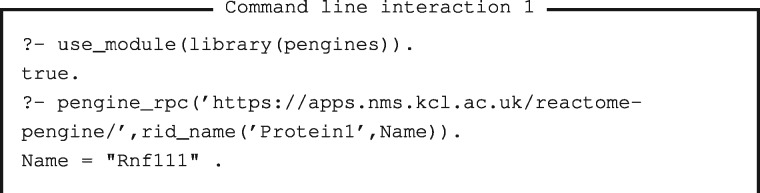


The second more advanced example shows how to send a program (alongside a query that calls the program) to Reactome Pengine to be computed remotely. For instance, a bioinformatician can use Reactome Pengine to explore paths of reactions through the human reactome. Code block 1 shows an example Prolog program that can be used for this purpose (available on Github at https://github.com/samwalrus/reactome-pengine). This program includes two core elements. First, the predicate *path_program/1* retrieves a list of clauses that themselves define a program that will be sent to the Reactome Pengine. Second, the predicate *path_from_to/3* is the main predicate that a bioinformatician would use to query the Reactome for paths in a variety of ways (without downloading the entire dataset to their machine). For instance, a researcher can use this predicate to (i) establish whether a path exists from a particular reaction to another, (ii) retrieve all paths from a reaction or (iii) retrieve all paths to a reaction. The *path_from_to/3* predicate first retrieves the Reactome Pengine server address (line 25) and the program (specified in *path_program/1* lines 4-23 and called on line 26) and then sends this program alongside a specified query to Reactome Pengine (lines 27-29). Command line interaction 2 shows example commands that use this program. Furthermore, notebook examples 6 and 7 show additional refinements to this program, such as further constraints for properties of reaction paths.

**Code Block 1.**
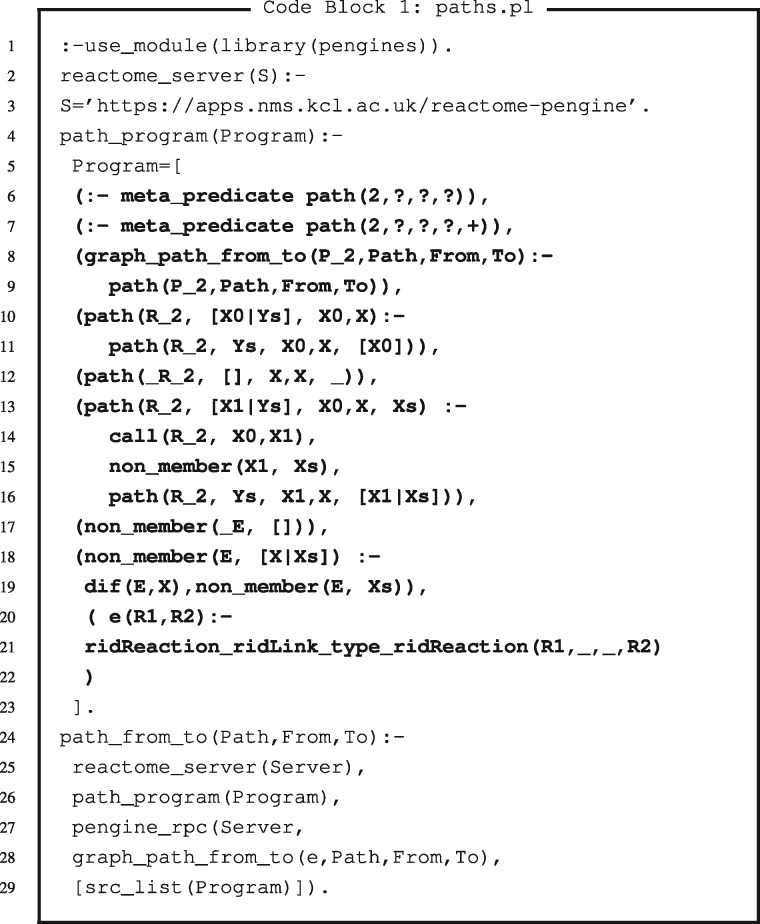
Bold text indicates the program sent to Reactome Pengine. In this example, the program is a list of terms, where each term is a clause that will be interpreted by Reactome Pengine. Adapted from https://stackoverflow.com/questions/30328433/definition-of-a-path-trail-walk/30595271#30595271


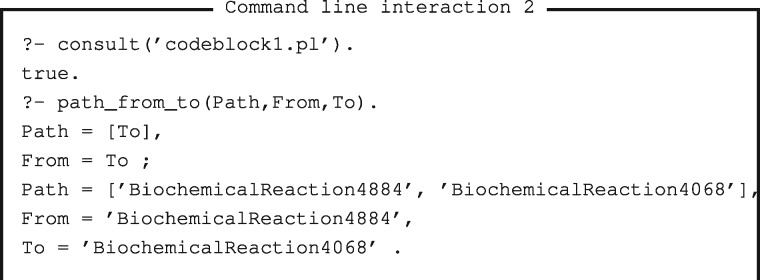


## 5 Summary

The Reactome Pengine is a web service that provides a simple way to logically query the human reactome on the web. It can be accessed by both local Prolog programs and web notebooks such as SWISH. The Pengine technology allows the user to send the ‘small’ program to the ‘large’ data. Increasingly more (and larger) biological datasets are becoming available online. While we have presented a Pengine web service for Reactome, it is possible to build these for any other online biological dataset. This is potentially very powerful, as researchers will not have to download and manage these datasets but can build pipelines that consist of a set of programs sent to these pengine web services.

## Funding

LACM is funded by the UK Medical Research Council [grant no. MC_UU_12013/8] and a University of Bristol Vice-Chancellor’s Fellowship.

*Conflict of Interest*: none declared.

## Supplementary Material

Supplementary Data
