# Supplementary material for: Reactome Pengine: a web-logic API to the Homo sapiens reactome
Source: Bioinformatics. 2018 Mar 30;34(16):2856–8. doi: 10.1093/bioinformatics/bty181 (PMC6084589; doi:10.1093/bioinformatics/bty181)
Supplement: Supplementary Data [file bty181_supplementary_information.pdf]

Reactome Pengine : Supplementary Information

S.R. Neaves, S.Tsoka, L.A.C. Millard

March 31, 2018

1 Reactome Pengine data flows

Figure 1 illustrates the flow of data between the users computer and Reactome Pengine, which could occur either directly or through SWISH. To use Reactome Pengine directly (shown as yellow/dashed arrows in Figure 1) the user writes a local Prolog program containing a subroutine that sends a query or program to Reactome Pengine. Reactome Pengine executes the program and returns data back to the local calling program to finish its execution.

To use SWISH to interact with Reactome Pengine (shown as grey/solid arrows in Figure 1) the user writes a program (program A in Figure 1) for SWISH, that will itself contain a query or program (program B in Figure 1) to be processed on Reactome Pengine. When SWISH executes program A, the constituent program B is forwarded to Reactome Pengine. Upon receiving program B Reactome Pengine executes it and sends the results back to SWISH. SWISH continues program A and then displays the results in the user's browser.

An example SWISH notebook that uses Reactome Pengine can be found at: `https://apps.nms.kcl.ac.uk/reactome-pengine/`. An example local Prolog script that uses Reactome Pengine is given in Supplementary Section S3.

2 Comparison of Reactome Pengine to existing data access options

This section compares the use of Reactome Pengine to: 1) downloading the entire dataset directly from Reactome and 2) downloading subsets of the data using the existing Reactome Application Programming Interfaces (APIs). We compare 1) the amount of data exchanged between the user's computer and Reactome Pengine, and 2) the degree of flexibility of querying the data.

2.1 Amount of data exchanged

We compare the amount of data exchanged between the user's machine and Reactome Pengine in contrast with downloading the complete dataset from

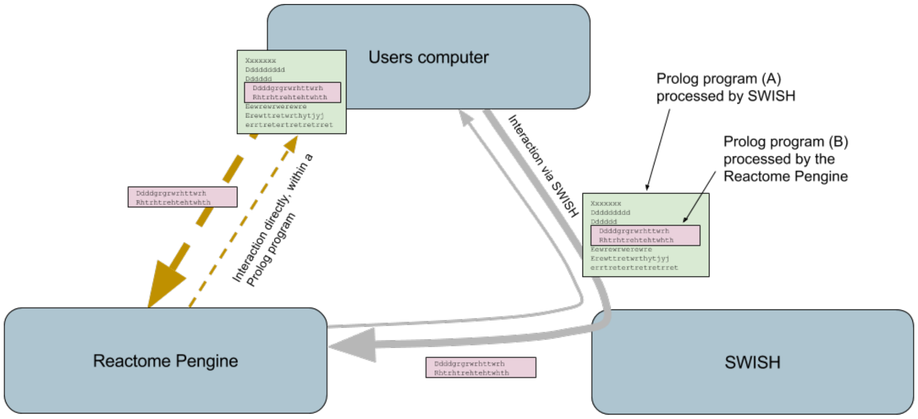

Figure 1: Diagram of Reactome Pengine. Thick arrows: data sent to Reactome Pengine; thin arrows: data returned from Reactome Pengine. Yellow, dashed: Direct interaction with Reactome Pengine; grey, solid: Interaction with Reactome Pengine via SWISH

Reactome and working with a local copy of the data. Reactome Pengine is built on the biopax RDF file (Homo_sapiens.owl) available at reactome `http://www.reactome.org/pages/download-data/`. Therefore, when not using Reactome Pengine the amount of data transferred when downloading this data to the user's machine is just the size of this file, which is 136.6 MB. It is also possible to download the dataset in different formats, such as CSV files, an SQL database or a Neo4J graph database, each with there own storage requirements.

The amount of data transferred when using Reactome Pengine is dependent on the query or program that the user submits. As Reactome Pengine is designed for non-trivial but not intensive use of the Reactome data, typically short programs will be sent to server. We give an example program in Code Block 1. This program is able to find paths of reactions in the reactome, which is a more complex query than the REST API can complete, but one that can easily be performed without downloading the whole Reactome dataset, using Reactome Pengine. The size of the data exchanged using this query is in the order of kilobytes.

As described in the main paper, Reactome Pengine is intended as a pioneering application that demonstrates how pengine technology is useful for bioinformatics research. While downloading the Reactome dataset in its entirety is currently feasible, biological datasets will only increase in size, such that efficient and flexible data querying approaches, such as pengines, will be imperative for future analysis involving the integration of omics data.

Code Block 1

```
:-use_module(library(pengines)).
reactome_server("https://apps.nms.kcl.ac.uk/reactome-pengine").
path_program(Program):-
 Program=[
 (:- meta_predicate path(2,?,?,?)),
 (:- meta_predicate path(2,?,?,?,+)),
 (graph_path_from_to(P_2,Path,From,To):-
    path(P_2,Path,From,To)),
 (path(R_2, [X0|Ys], X0,X):-
    path(R_2, Ys, X0,X, [X0])),
 (path(_R_2, [], X,X, _)),
 (path(R_2, [X1|Ys], X0,X, Xs) :-
    call(R_2, X0,X1),
    non_member(X1, Xs),
    path(R_2, Ys, X1,X, [X1|Xs])),
 (non_member(_E, [])),
 (non_member(E, [X|Xs]) :-
  dif(E,X),non_member(E, Xs)),
  ( e(R1,R2):-
  ridReaction_ridLink_type_ridReaction(R1,_,_,R2)
  )
 ].
path_from_to(Path,From,To):-
 reactome_server(Server),
 path_program(Program),
 pengine_rpc(Server,
 graph_path_from_to(e,Path,From,To),
 [src_list(Program)]).
```

Figure 2: Adapted from Stack Overflow definition of a path/trail/walk. Accessed: 2017-10-21. https://stackoverflow.com/questions/30328433/definition-of-a-path-trail-walk/30595271#30595271 .

2.2 Flexibility of querying

In this section we discuss the increased flexibility of using Reactome Pengine compared to existing data access options.

2.2.1 Reactome Pengine versus existing Reactome APIs

We compare Reactome Pengine with 1) the REST API and 2) the SPARQL API. First, the REST API is limited to a number of queries designed by the Reactome maintainers (see documentation here http://www.reactome.org/pages/documentation/developer-guide/restful-service/#API). Any query that can be performed using the REST API, can also be performed using Reactome Pengine. For example, the REST service can be used to find the subpathways of ‘Apoptosis’, and an equivalent query using Reactome Pengine is given in Code Block 2. We invoke the query with:

```
?-pathwayName_subpathway('Apoptosis',Subpathway).
```

In contrast to the REST API we can build upon this query to create composite queries. For example, we could add further constraints to the query to find sub-pathways with particular properties.

Code Block 2

```
:-use_module(library(pengines)).
reactome_server('https://apps.nms.kcl.ac.uk/reactome-pengine').

my_program(P):-
    P=[
      (
      pathwayName_subpathway(PName,SubName):-
          rid_name(RidPathway,PName),
          ridPathway_component(RidPathway,RidComponent),
          rid_type_iri(RidComponent,'Pathway',_),
          rid_name(RidComponent,SubName)
      )

      ].

pathwayName_subpathway(PName,SubName):-
    reactome_server(S),
    my_program(P),
    pengine_rpc(S,pathwayName_subpathway(PName,SubName),[src_list(P)]).
```

Any SPARQL query can be performed using Reactome Pengine. For example the SPARQL documentation from Reactome gives an example query that finds pathways that have entities in the cellular membrane (`https://www.ebi.ac.uk/rdf/documentation/reactome/`). The Prolog program in Code Block 3 uses Reactome Pengine to perform this query.

The Reactome SPARQL API is more flexible than the REST API for two key reasons. First, SPARQL can be used to specify SQL like queries over the data, rather than a predefined subset specified by an API. Second, SPARQL can be used to interrogate several datasets in a single query (called a federated query). For example, a bioinformatician could query both Reactome and Uniprot to integrate data from these disparate sources.

While SPARQL is more flexible than a REST API, it is less flexible than Reactome Pengine because it is not a full programming language. This means that typically developers using SPARQL will have a two language setup, for example, SPARQL might be embedded in Java. This can be problematic due to the paradigm mismatch, where SPARQL is relational and Java is object oriented. This is not the case for Prolog which has a relational paradigm itself, and is a full programming language. Therefore, using Reactome Pengine from within a Prolog program means that the data can be queried and manipulated within a single program.

The Reactome Pengine Prolog API allows for simpler and more flexible federated queries than using SPARQL. Complex federated queries are simpler to compose in the Reactome Pengine due to its ability to build composite queries, as discussed above. Furthermore, because we can make use of standard Prolog libraries within the query sent to the Reactome Pengine, we can also include queries to other data services, including REST, SPARQL, HTML and other pengine services. An example of this is given in the accompanying SWISH notebook (example 9).

Code Block 3

```
:-use_module(library(pengines)).
reactome_server('https://apps.nms.kcl.ac.uk/reactome-pengine').

program(P):-
    P=[
      (
       pathway_acrossmembrane(Pathwayname):-
          Location = "plasma membrane",
          rid_type_iri(RidPathway,'Pathway',_Iri),
          rid_name(RidPathway,Pathwayname),
          ridPathway_component(RidPathway,RidReaction),
          ridReaction_input(RidReaction,RidEntity),
          rid_location(RidEntity,Location)
      )
      ].

pathway_acrossmembrane(PathwayName):-
    reactome_server(S),
    program(P),
    pengine_rpc(S,pathway_acrossmembrane(PathwayName),[src_list(P)]).
```

2.2.2 Reactome Pengines data access predicates

In addition to providing the data available from Reactome, Reactome Pengine also includes over 30 public predicate definitions which offer intuitive and fast access to elements of the data. Full details of these predicates are available in the online documentation `https://apps.nms.kcl.ac.uk/reactome-pengine/documentation`. Furthermore, as Reactome Pengine is monitored, commonly used predicates can be added to Reactome Pengine.

2.2.3 Reactome Pengines querying language

It is also possible to have the web-logic query embeded in another language that supports http requests. For example a shell script, java script or python. Code Block 4 gives an example of a node js program that uses the pengines npm module `https://www.npmjs.com/package/pengines`.

Code Block 4

```
pengines = require('pengines');

peng = pengines({
    server: "https://apps.nms.kcl.ac.uk/reactome-pengine/pengine",
    sourceText: 'small_pathway(P):- ridPathway_links(P,L), length(L,S), S<35.',
    ask: "small_pathway(X)",
    chunk: 100,
}
).on('success', handleSuccess).on('error', handleError);
function handleSuccess(result) {
    console.log(result)
}
function handleError(result) {
    console.error(result)
}
```

This feature is useful when introducing Reactome Pengine to existing code pipe lines that might not be written in Prolog. However building Prolog pipelines and using Prolog as the 'glue' language is very powerfull as we have illustrated throughout this work. Notablly the abililty to 'name and reuse' queries (exam-

ple 5 in the accompanying notebook https://swish.swi-prolog.org/?code=https://raw.githubusercontent.com/samwalrus/reactome_notebook/master/reactome_pengine.swinb) and the fact that Prolog is a Homoiconic language (where data and code use the same syntax) means that Prolog pipe lines are very effective for bioinformatic work - especially for queries across multiple data end points (sometimes known as fedderated queries).

2.2.4 Reactome Pengines data output format

It is possible to change the format of replies from the Reactome Pengine from Prolog terms to either CSV or JSON file formats. For an example of a shell script that queries a pengine for a csv file see: https://github.com/SWI-Prolog/swish/blob/master/client/swish-ask.sh

3 Example Prolog script for UNIX pipeline

As discussed in the main paper, Reactome Pengine can be used directly in a Prolog program on a local machine (Tested on SWI-Prolog version 7.7). An example Prolog script is given in Code Block 5, which can be used in a general UNIX pipeline (see for example Code Block 6). This script takes a file with a Reactome protein identifier on each line and outputs the affymetrix probe identifiers for each protein. In order to execute this script the user will need to make the script executable using the UNIX command 'chmod'.

Code Block 5

```
#!/usr/bin/env swipl

:- use_module(library(pengines)).
:- initialization main.

server(S):-S="https://apps.nms.kcl.ac.uk/reactome-pengine".

main :-
        catch(readloop, E, (print_message(error, E), fail)),
        halt.
main :-
        halt(1).


readloop:-
        read_line_to_string(user_input,String),
        string_test(String).

string_test(String):-
        dif(String,end_of_file),
        atom_string(Atom,String),
        ridProtein_probelist(Atom,Animal),
        writeln(Animal),
        readloop.

string_test(Term):-
        Term = end_of_file,
        fail.


ridProtein_probelist(R,P):-
    server(S),
    pengine_rpc(S,ridProtein_probelist(R,P),[]).

```

Example File: proteins.txt

```
Protein56
Protein17
Protein34
```

:

Code Block 6: Example UNIX pipeline

```
./codeblock5.pl < proteins.txt | grep 1565484_x_at
```
